# From marine park to future genomic observatory? Enhancing marine biodiversity assessments using a biocode approach

**DOI:** 10.3897/BDJ.7.e46833

**Published:** 2019-12-10

**Authors:** Yin Cheong Aden Ip, Ywee Chieh Tay, Su Xuan Gan, Hui Ping Ang, Karenne Tun, Loke Ming Chou, Danwei Huang, Rudolf Meier

**Affiliations:** 1 Department of Biological Sciences, National University of Singapore, Singapore, Singapore Department of Biological Sciences, National University of Singapore Singapore Singapore; 2 National University of Singapore, Singapore, Singapore National University of Singapore Singapore Singapore; 3 Temasek Life Sciences Laboratory, Singapore, Singapore Temasek Life Sciences Laboratory Singapore Singapore; 4 National Parks Board, Singapore, Singapore National Parks Board Singapore Singapore; 5 Tropical Marine Science Institute, National University of Singapore, Singapore, Singapore Tropical Marine Science Institute, National University of Singapore Singapore Singapore

**Keywords:** DNA barcoding, marine park, genomic observatory, COI, biocodes

## Abstract

Few tropical marine sites have been thoroughly characterised for their animal species, even though they constitute the largest proportion of multicellular diversity. A number of focused biodiversity sampling programmes have amassed immense collections to address this shortfall, but obstacles remain due to the lack of identification tools and large proportion of undescribed species globally. These problems can be partially addressed with DNA barcodes (“biocodes”), which have the potential to facilitate the estimation of species diversity and identify animals to named species via barcode databases. Here, we present the first results of what is intended to be a sustained, systematic study of the marine fauna of Singapore’s first marine park, reporting more than 365 animal species, determined based on DNA barcodes and/or morphology represented by 931 specimens (367 zooplankton, 564 macrofauna including 36 fish). Due to the lack of morphological and molecular identification tools, only a small proportion could be identified to species solely based on either morphology (24.5%) or barcodes (24.6%). Estimation of species numbers for some taxa was difficult because of the lack of sufficiently clear barcoding gaps. The specimens were imaged and added to “Biodiversity of Singapore” (http://singapore.biodiversity.online), which now contains images for > 13,000 species occurring in the country.

## Introduction

In recent decades, it has become clear that biodiversity loss is an increasingly serious problem and many species are expected to become extinct before discovery and description (Costello et al. 2013, Laurance 2013). It is thought that only 226,000 of the estimated 0.7–1 million marine species have been described (Appeltans et al. 2012). Poor sampling of marine fauna in biodiverse regions and a large backlog of species that have yet to be described have rendered most marine species unidentifiable and often unknown to science (Mora et al. 2011, Mora et al. 2013). This incomplete knowledge of species diversity prevents accurate biodiversity assessments and monitoring and limits our understanding of ecosystem functioning (Isaac et al. 2004). Determining species diversity, using traditional taxonomic techniques, requires skilled taxonomists to accurately identify or describe species based on detailed keys and careful study of morphology. However, this approach is manpower-intensive, slow (Miller 2007) and costly (approximately $39,000–122,000 per species; Carbayo and Marques 2011). Consequently, alternative strategies are being developed to expedite the processes of species discovery and delimitation (Wang et al. 2018). While convincing solutions for large-scale species description are lacking, the problem is starting to attract the attention of many animal taxonomists (Riedel et al. 2013).

Molecular techniques have dramatically increased the rate of species discovery and facilitated species identification for those species that have been barcoded. DNA barcoding was initially proposed as a means to identify animal species, although it is now increasingly used for species discovery (Hebert et al. 2003, Ratnasingham and Hebert 2007, Hajibabaei et al. 2007, Goldstein and DeSalle 2011). This technique uses a short DNA fragment as a standard marker for species description and discovery. For metazoans, the mitochondrial cytochrome c oxidase subunit I (COI) is the barcoding locus of choice, having been popularised by Hebert et al. 2003. However, as a prerequisite to successful species identification via barcodes, a comprehensively curated reference database is required (Ekrem et al. 2007). Public databases, such as GenBank (Benson et al. 2018) and the Barcode of Life Data (BOLD) System (Ratnasingham and Hebert 2007), collect such reference sequences and, while they contain many sequences for vertebrates (Hebert et al. 2004, Ward et al. 2005, Kerr et al. 2007), the coverage for invertebrate species is more limited (Barrett and Hebert 2005, Hajibabaei et al. 2006, Elias et al. 2007, Grant and Linse 2009, Hausmann et al. 2011, Park et al. 2011). These databases are continually updated as new discoveries are made and presently the BOLD System contains > 5 million barcode sequences belonging to 262,679 species (as of December 2017). However, these identifications should be interpreted with caution, because many are for predicted species (Barcode Index Numbers; “BINS”) and few specimens have been formally identified or verified as species (Kwong et al. 2012). Despite these potential issues, research is uncovering unexpected diversity for many taxa in many habitats (Hebert et al. 2004, Brower 2006, Witt et al. 2006, Havermans et al. 2011). These discoveries further reinforce the utility of DNA barcoding in surveys of biodiversity (Smith et al. 2005).

For highly biodiverse regions such as Southeast Asia, these global reference databases remain particularly incomplete and poorly curated (Giam et al. 2010, Jinbo et al. 2011), partially due to the prohibitive costs associated with molecular sequencing (Meier 2008). The sheer number of species found in biodiversity hotspots also poses a considerable challenge, as many of the barcodes recovered differ from those in the databases by more than 3%, meaning accurate species identification is not possible for these animals (Kwong et al. 2012) and several of which may also be new species (Wong et al. 2011, Wang et al. 2018). These problems will only be resolved by greater sampling effort and expansion of curated databases. Unfortunately, a shortage of taxonomic expertise for biodiverse regions is compounded by a lack of the necessary skills required to perform the field collections, a consequence of the decline in biodiversity appreciation (Ríos-Saldaña et al. 2018). It is here that digital reference collections, such as “Biodiversity of Singapore” (https://singapore.biodiversity.online/), can make a difference by helping to stimulate an interest in biodiversity and conservation, with verifying putative species identifications (Will et al. 2005, Ang et al. 2013, Chan et al. 2014).

Singapore is situated just outside the southwest corner of the biodiverse Coral Triangle biodiversity hotspot and, like other countries in the region, its marine biodiversity remains relatively poorly understood. To address this shortfall, we describe the first results of a programme that aims to build a comprehensive animal species identification database for Singapore’s first marine park—the Sisters’ Islands Marine Park (SIMP; Fig. 1). Prior to this study, preliminary estimates suggested that the SIMP may be home to > 100 fish and > 1,000 macroinvertebrate species (K. Tun, pers. obs.). Barcoding the fauna is part of a larger initiative to make Singapore’s biodiversity identifiable with molecular tools and constitutes the first steps towards building a national genomic observatory (Davies et al. 2014). Located approximately 6 km south of mainland Singapore, SIMP is recognised as a locally important area of biodiversity in terms of coral species richness, functional and phylogenetic diversity (Wong et al. 2018). Previous work modelling coral larval dispersal has indicated that reefs in the area are potentially strong source reefs that can seed other reefs in Singapore (Tay et al. 2012, Chang 2015). Furthermore, the marine park also aims to serve as an outreach platform to encourage public interest in marine life and participation in biodiversity conservation. It is open to the public and visitors can interact with the natural environment via guided intertidal walks and subtidal dive trails. Thus, a systematic and regular documentation of the biodiversity at the SIMP is important for the management and conservation of marine ecosystems in Singapore. SIMP was established as a marine park on 15 July 2015 and spans an area of 40 hectares that encompasses the namesake Sisters’ Islands (Pulau Subar Laut and Pulau Subar Darat), the western shores of Pulau Tekukor and Pulau Sakijang Bendera (Fig. 1).

The work performed here will help consolidate sampling records and molecular data obtained from the SIMP will form an important baseline for monitoring Singapore’s marine species. It will also provide better and more complete understanding of marine biodiversity in Singapore, with further utility throughout Southeast Asia where work of this nature is still in its infancy and which is inadequately represented in global databases (Koh and Sodhi 2010, Webb et al. 2010). Finally, it serves as a resource for future work relying on curated databases for species detection and discovery of species interactions, such as environmental DNA (eDNA) (Ficetola et al. 2008, Jerde et al. 2011, Thomsen et al. 2011, Yamamoto et al. 2017, Djurhuus et al. 2018). Given the rapid increase in interest in using molecular data for environmental monitoring in recent years (Harper et al. 2018, Darling 2019, Rey et al. 2019), these will likely have crucial applications for the conservation and management of marine resources.

## Materials and Methods

### Literature review

We first compiled existing records and published DNA barcodes relevant to SIMP through a literature keyword search for marine fauna found at the SIMP. Records for SIMP species predominantly came from two survey projects aimed at documenting and/or discovering local biodiversity (without DNA barcodes): (i) a large-scale ‘Comprehensive Marine Biodiversity Survey’ of Singapore (Tan et al. 2013), which sampled marine fauna across Singapore using many different sampling methods, from hand sampling to dredging and (ii) a BioBlitz initiative by the National Parks Board of Singapore, which is a series of visual surveys involving volunteer scientists and members of the public (Suppl. material 1) that documented, for example, 105 scleractinian coral species at the SIMP. Barcode data for some of the species on the list were obtained via GenBank (109 species; Suppl. material 2) and added to the SIMP database.

### Macrofauna

#### Field collection

Samples were collected from all four islands of the SIMP:

1. Pulau Subar Laut (Big Sister’s Island; 1.21417°N, 103.83444°E),
2. Pulau Subar Darat (Small Sister’s Island; 1.215788°N, 103.832705°E),
3. Pulau Sakijang Bendera (specifically Tanjong Hakim; 1.213823°N, 103.851107°E) and
4. Pulau Tekukor (1.232139°N, 103.836604°E).

Collections were authorised by the National Parks Board (permit number NP/RP15-088) and were carried out at the accessible intertidal reef, sandy beach, seawall and much of the shallow subtidal reef areas (Fig. 1) over a span of two years from July 2015 to July 2017.

Intertidal specimens were obtained using hand tools and nets during low spring tides, 0.0 m to 0.2 m above chart datum. These tools were likewise used for subtidal sampling via SCUBA diving to depths of up to 15 m. The search included around, under and inside potential hideouts. Any metazoans encountered during these visual surveys that were not already in our collection, were collected. Fish were collected using two ‘bubu’ traps, each measuring 0.072 m^3^, deployed twice, for periods of one day each, during the sampling period. Up to three individuals of each species were collected, avoiding gravid females and juveniles to reduce sampling impact on natural populations.

#### Sample processing and imaging

Samples were provisionally imaged *in situ* using a Canon Powershot G10 (Canon Inc., Japan) or Olympus Stylus Tough TG-4 compact camera (Olympus Corporation, Japan). In the laboratory, invertebrate specimens were relaxed in 7.5% (w/v) MgCl_2_ buffered in seawater (Messenger et al. 1985), while fish specimens were handled according to NUS Institutional Animal Care and Use Committee (IACUC) guidelines (IACUC Protocol B15-1403). A Canon EOS 750D or a dissecting microscope (Leica S8 APO with Canon EOS 750D mounted; 1–8× magnification) was used for specimen imaging. Tissue subsamples were then taken from each specimen before fixation or preservation. Hard-bodied specimens were preserved in 70% (v/v) molecular grade ethanol, while soft-bodied organisms were first fixed in 4% (v/v) formaldehyde overnight, then transferred to 70% ethanol for long-term preservation. All macrofaunal vouchers were deposited at the Zoological Reference Collection (ZRC) of the Lee Kong Chian Natural History Museum (LKCNHM) as voucher specimens (Suppl. material 3) and the available image data made available online at the “Biodiversity of Singapore”, a digital reference collection for Singapore’s biodiversity (http://singapore.biodiversity.online; Fig. 2).

#### Tissue subsampling, digestion and DNA extraction

For each large soft-bodied specimen, a small piece of tissue (20–40 mm3) was excised, while for each arthropod, one to two legs from the same side of the body were detached for DNA extraction. The tissues were digested overnight at 55°C in 900 μl CTAB (hexadecyltrimethylammonium bromide) with 0.4 mg proteinase K, after which DNA was purified by phase separation with phenol: chloroform: isoamyl-alcohol (25:24:1).

#### COI barcode amplification

The COI gene region was amplified using different primer pairs described in Folmer et al. 1994, Leray et al. 2013, Lobo et al. 2013, henceforth referred to as the ‘Folmer’, ‘Leray’ and ‘Lobo’ primers, respectively. Reactions were performed using one of three mixes:

1. BioReady rTaq DNA polymerase, 1× reaction buffer (v/v) (Bulldog Bio Inc., China) with the Folmer primer pair targeting the 658-bp barcode region of the COI gene;
2. GoTaq® DNA polymerase (Promega Corporation, U.S.A.) with the Lobo primer pair amplifying the same 658-bp COI region; or
3. GoTaq® Green Master Mix (Promega Corporation, U.S.A.) with the ‘Lobo reverse and Leray forward’ primer combination targeting a shorter 313-bp COI region for samples that were particularly challenging to amplify.

Most of the macrofaunal samples were subjected to Sanger barcoding. Each 12.5-μl reaction contained 0.5 μM of each primer (uniquely tagged primers for 46 samples only; untagged for the rest), 0.5 μg BSA (bovine serum albumin), 2 μl template DNA and 1× GoTaq®/BioReady rTaq DNA polymerase and reagents mastermix (v/v), according to the manufacturer’s recommendations. The thermal cycling profile for (1) using the Folmer primer pair was 94°C for 60 s; 35 cycles of denaturation at 94°C for 45 s, annealing at 48°C for 45 s, extension at 72°C for 90 s; and a final extension at 72°C for 3 mins. The thermal cycling profile for (2) and (3), using the Lobo primers, included a step-up annealing profile of 94°C for 60 s; 5 cycles of 94°C for 30 s, 48°C for 120 s, 72°C for 60 s; 35 cycles of 94°C for 30 s, 54°C for 120 s, 72°C for 60 s; and 72°C for 5 mins.

#### DNA barcoding using Sanger barcoding

Successful PCR amplicons were purified using SureClean Plus (Bioline Inc., London, UK) and prepared for Sanger sequencing using the BigDye Terminator Cycle Sequencing Kit v. 1.1 and PureSEQ (Aline Biosciences), on an Applied Biosystems 3730XL DNA Analyzer (Thermo Fisher Scientific, U.S.A.), following the manufacturer’s instructions. COI barcodes, obtained via Sanger sequencing, were assembled and edited using Geneious R11 v11.0.2 (Biomatters Limited) (Kearse et al. 2012). Although the cost of generating DNA barcodes with Sanger sequencing is expensive (Meier et al. 2015), it was used for barcoding most macrofaunal samples since collections were conducted in numerous small batches (Suppl. material 3) that were too small for cost-effective barcoding via high-throughput sequencing (HTS).

### Zooplankton

#### Field collection

Sampling was performed at sites 1, 3 and 4 listed in the macrofaunal field collection section. A vertical plankton tow with a 100-μm mesh net was used to collect micro- and mesozooplankton (Sieburth et al. 2003) upwards from a depth of 8 m. Zooplankton were concentrated into a 50-ml bottle of seawater from each tow, put on ice and brought back to the laboratory for processing.

#### Sample processing and imaging

Samples were concentrated through a 100-μm sieve, preserved in 70% ethanol and stored at -30°C prior to sample sorting and imaging. Sorting and imaging were performed under a dissecting microscope (Leica S8 APO with Canon EOS 750D mounted; 1–8× magnification), using soft fine forceps. Specimen identification followed Johnson and Allen (2005) and samples were preliminarily grouped into seven morphotypes at the phylum level (Arthropoda, Annelida, Chaetognatha, Chordata, Cnidaria, Mollusca and Platyhelminthes). Each zooplankton was processed and stored individually in 70% ethanol in 96-well plates at -30°C. Zooplankton identities were later confirmed using DNA barcodes.

#### Tissue subsampling, digestion and DNA extraction

For larger arthropods, one or two legs from the same side of the body were detached for DNA extraction. For specimens < 5 mm in size, whole individuals were either used for phenol-chloroform extraction or were incubated in 20 μl of 2×-diluted QuickExtract TM DNA extraction solution (Epicenter, BuccalAmp TM) , following the manufacturer’s instructions.

#### COI barcode amplification

Forty-six macrofaunal samples, along with all zooplankton samples, were sequenced using high-throughput sequencing (HTS; Suppl. material 4). PCR was performed on genomic DNA extracted using QuickExtract (Epicenter, BuccalAmpTM) or directly on selected samples for improved time efficiency (Wong et al. 2014). A short 313-bp fragment of the COI gene was targeted using either the mlCO1intF and rmHCO2198 primer pair (Folmer et al. 1994, Meier et al. 2015) or reaction mix (3) (see section on macrofaunal COI barcode amplification) with forward and reverse primers that were uniquely labelled with 9-bp tags (generated with online freeware “Barcode generator”; http://comailab.genomecenter.ucdavis.edu/index.php/Barcode_generator) that differed from one another by ≥ 3-bp (Meier et al. 2015). Each 20-μl PCR reaction contained 1× GoTaq® Green Master Mix, 0.5 μM of each uniquely labelled primer and 2 μl of DNA extract. PCR thermal cycling conditions were as follows: an initial denaturation step at 94°C for 60 s, followed by 35 cycles of 94°C for 60 s, 47°C for 120 s, 72°C for 60 s, and 72°C for 3 mins.

#### DNA barcoding using high-throughput sequencing

DNA barcoding via HTS ("HTS barcoding"; Wang et al. 2018) can be used to process a large number of specimens (e.g. zooplankton from bulk samples), using a reverse workflow where all specimens are barcoded and pre-sorted into MOTUs that are considered putative species awaiting verification by taxonomic experts (Wang et al. 2018, Gan et al. 2019). HTS barcoding is faster and more than one order of magnitude cheaper than Sanger barcodes (Meier et al. 2015). Tagged amplicons were pooled into four libraries (NEBNext ® UltraTM II DNA Library Prep) for sequencing over five lanes of the Illumina MiSeq platform (v3; 2 × 300 bp; 25 million single reads). Note that these samples only took up 0.2% to 1.6% of each lane. HTS COI barcodes obtained via Illumina MiSeq sequencing were retrieved following the pipeline described in Meier et al. 2015. Briefly, paired-end read data were assembled using PEAR version 0.9.6 (Zhang et al. 2013), data for individual samples were demultiplexed and dominant read sets per sample were identified. To ensure an accurate barcode database, the data were subsequently filtered for sequencing coverage > 50, then filtered by a total barcode count of > 10 and finally against potential contamination by retaining data where the dominant read set was at least four times as abundant as the second dominant read set for each sample (i.e. ratio of coverages of second: first dominant read ≤ 0.2). Samples, for which this ratio was < 0.35, were further evaluated to assess if their sequences could still be used (i.e. if barcodes were consistent with morphology).

#### Matching zooplankton barcode identities to morphotype data

Four criteria (C1–4) were used to select barcodes that we considered reliable. C1: Zooplankton morphotypes and barcode identities were congruent and samples had a good match (≥ 97%; giving species level identity) to global databases. C2: Barcode had a poor match (> 85%; giving lowest taxonomic identity), but the match was consistent with the morphological sort. C3: In order to accommodate mistakes that may be made during the initial sort of zooplankton, we kept sequences for specimens, even when the morphotypes and barcode identities were incongruent as long as the BLAST match to an existing species in GenBank was high (≥ 97%) and the specimen images were consistent with the BLAST matches. C4: Specimens that failed to yield a barcode due to the violation of filtering thresholds were re-evaluated and retained when all of the following criteria were fulfilled:

- The ratio of first to second dominant read was 0.2–0.35;
- Sequencing coverage of > 50 reads and total barcode count of > 10;
- The dominant read was ≥ 85% match to a taxon that was congruent to morphotype data (see C1); and
- The second dominant read did not match the preliminary assigned morphotype.

### Sequence data analysis

All sequence data were aligned using MUSCLE 3.8.425 (Edgar 2004), translated and screened for stop codons using Geneious R11 v11.0.2 (Biomatters Limited) (Kearse et al. 2012). Only one specimen, *Peronia verrculata* (IP0136), was found to have a deletion of one codon compared to all other sequences. This deletion was confirmed against 63 other *P. verruculata* specimens previously collected in Singapore (Chang et al. 2018). Data were subject to a final round of contamination check by comparing BLAST (Altschul 1990) matches against GenBank (Benson et al. 2018) and the Barcode of Life Data (BOLD) System (Ratnasingham and Hebert 2007), for consistency with specimen morphology. BLAST matches were used to identify the specimens where query cover was ≥ 80%. The thresholds used were ≥ 80% identity for family, ≥ 90% identity for genus and ≥ 97% identity for species identification. It must be noted that these identities at family and genus levels were conservative, based on general interspecific distances which have been observed amongst various marine taxa (Sun et al. 2016, Trivedi et al. 2015), but should be treated with caution as supraspecific ranks are not consistently defined (Bertrand et al. 2006, Kuntner and Agnarsson 2006). Finally, an objective clustering method, with internal gaps treated as a fifth character, was used to group sequences into MOTUs, based on variable pairwise sequence similarities (Srivathsan, unpublished software; implementation of objective clustering described in Meier et al. 2006). One of the barcodes (IP0303), was excluded from this step due to a large internal gap that interferes with clustering.

## Results

Sampling at Sisters’ Islands Marine Park (SIMP) yielded 931 specimens, comprising 564 macrofauna (benthic and fish) and 367 zooplankton specimens (Figs 3, 4, Suppl. materials 3, 4). Of all the specimens, 24.5% were identified by morphology to species, based on literature. They were included in a database of ca. 150 species that was assembled prior to barcoding. Most of the sampled fauna belonged to arthropods (38.9% of 931 samples) and molluscs (21.0%), followed by cnidarians (9.5%) and annelids (8.3%) (Fig. 5). Fish were the least represented, with only 36 specimens (3.9%).

Overall, COI amplification success was 68.0% across all phyla (633 out of 931 samples). A total of 297 of the sample barcodes (46.9%) were ≥ 658-bp in length (long; average length 677-bp), while 336 samples (53.1%) had sequence lengths varying between 229- and 657-bp (short; average length 350-bp) (Suppl. materials 3, 4). No deletions, insertions or stop codons were observed in any of the COI sequences, suggesting that the data did not include nuclear DNA sequences, originating from mitochondrial DNA (NUMTs) (Bensasson 2001).

Amplification and sequencing success were variable across different phyla and primer pair combinations. Molluscs were generally easy to amplify, while echinoderms were challenging and required more PCR optimisation. Specifically, primer pairs in reaction mix (1) yielded approximately 50% amplification success, reaction mix (2) gave approximately 80% success and reaction mix (3) yielded the highest amplification success at ≥ 95%.

PCR amplification success for zooplankton samples was 70.6% (of 367 samples) and 259 tagged amplicons were sequenced using high-throughput sequencing (HTS) barcoding. Due to uneven amplicon pooling, data for only 191 of these amplicons were retrieved, for which 411,201 reads were demultiplexed and sequence quality filters resulted in coverage of 19 to 4,048 reads per barcode. Overall, sequencing success was moderate, with 174 out of 259 samples (67.2%) passing all filtering criteria. Of those failing the criteria, nine specimens were retained following criteria C4.

Well-studied and morphologically distinct groups such as corals, sea anemones, echinoderms, molluscs and crustaceans were easily recognised, but most specimens could not be identified to species (75.3% unidentifiable; only 230 specimens were identifiable to 155 species by morphology) without DNA barcode-assisted identification. DNA barcodes obtained for 633 specimens clustered into 351–395 species dependent on clustering criterion (i.e. MOTUs), of which 83 specimens were identifiable only via DNA barcode (48 species). This adds up to an approximately 36% increase in the number of specimens that could be delimited to at least species level.

The final set of 633 COI specimen barcodes obtained clustered into 351 molecular operational taxonomic units (MOTUs, i.e. putative species). This was based on a species delimitation threshold of 3%, which was defined by assessing the percentage pairwise differences across all sampled taxon groups (Suppl. material 5). Clustering at 3% provides a conservative estimate of species, especially for Anthozoa—there may be up to 42 additional species of anthozoans and another species of *Costasiella* slug and synaptid sea cucumber (Suppl. material 5), bringing the final DNA barcode-based species count up to 395. Although barcodes for 305 of the 633 specimens were matched successfully (i.e. ≥ 97%) to global databases (Suppl. material 6), 49 of these matched to database sequences that were not identified to species (e.g. uncultured zooplankton and “sp.”), while another 28 were not congruent with our morphological identification. Hence, only 229 specimens (36.2% of 633) were successfully identified based on COI barcode matching alone, which represented 116 MOTUs/species at the 3% threshold. Morphological identification for 90 specimens without COI-based identification (< 97% identity match, ≥ 97% match to unidentified database sequences or mismatch to morphological identification) allowed for 304 specimen barcodes to be tagged with species names (48.0% of 633), which represented 149 species (Suppl. material 6). With the addition of a further 14 species, based on morphology alone, a total of 163 known species were identified across the 931 specimens collected at the SIMP. Ten of these were new (zooplankton) species records for Singapore. Including MOTUs not identifiable to species, we hereby report a total of more than 365 animal species collected from SIMP, based on morphological and/or genetic identifications. All sequence data have been deposited in GenBank (Accession numbers MN689967–MN690599) and BOLD.

## Discussion

In recent years, the process of species discovery has been enhanced with DNA barcoding approaches (Hebert et al. 2003). Relatedly, large-scale marine sampling programmes and expeditions such as the Moorea Biocode Project (Check 2006, Leray et al. 2011) and SANTO 2006 (Bouchet et al. 2009), focusing on South Pacific islands, have expedited species discovery and diversity estimation. Such biodiversity sampling expeditions are important, not just for documenting biodiversity; the massive collections also provide material for improving our understanding of the evolutionary histories of various groups (Holford et al. 2008,Redmond et al. 2013, da Silva Oliveira et al. 2017). In this study, nearly one thousand specimens belonging to more than 365 marine animal species were collected and processed over 24 months across 13 sites at the SIMP. These species represent ca. 300 macrofauna and 70 zooplankton species, which include ten new (zooplankton) species records for Singapore and 58 species that are hitherto not included or misidentified in two of the largest global COI barcode databases (GenBank and BOLD). While morphological identification of most groups could be performed to the family level, species-level sorting and identification was a key challenge before the use of DNA barcoding. This was demonstrated in morphologically distinct groups that were easily recognised, where DNA barcoding resulted in approximately 36% increase in the number of specimens that could be delimited to at least species level. These advances underscore the importance of COI barcoding, especially for large-scale biodiversity surveys.

The morphological study of small animals and zooplankton is particularly time-consuming because large numbers of specimens are usually collected (e.g. Schmoker et al. 2014, Gan et al. 2019). This includes planktonic larvae, which are some of the most difficult developmental stages to identify and are traditionally reliant on laboratory-reared larvae to match juveniles with adults (Miller et al. 1989, Johnson and Allen 2005). Barnacles (Cirripedia) in the water column have never been identified to species before (Schmoker et al. (2014), likely because their planktonic nauplii tend to be morphologically similar between species (Gaonkar et al. 2014). In the present study, a combination of conventional sorting via microscopy and barcode matching to the GenBank database yielded new zooplankton species records for Singapore within just two plankton-tow samples. In particular, adult-larva matching was achieved for four barnacle species in the plankton samples, as all could be successfully identified to species by their DNA barcodes. Furthermore, planktonic molluscs have high potential for phenotypic plasticity and have been shown to exhibit cryptic speciation, displaying substantial morphological overlap between species and distinct morphotypes in different life stages (Kocot et al. 2016, Sun et al. 2016). Being able to quickly associate larvae with adults using DNA barcodes is thus important to improve the sensitivity of species detection. Larvae typically provide a much larger pool of species’ signals that can increase the chances of species detection than with adults, which may often be rare or go undetected when adult females are collected. Expanding beyond the usual morphological work on adult male specimens through the use of DNA barcoding allows for a more comprehensive understanding of species’ ecology, such as their life histories and phenologies, since the life history stages of dozens of species can be cost-effectively matched in a single study (Yeo et al. 2018).

Morphological identification can be challenging, even for charismatic animals due to the presence of cryptic species. Our analyses revealed at least two pairs of morphologically indistinguishable species with high COI sequence divergence. These possibly sympatric cryptic species groups include two *Ligia* isopods with a 22% pairwise distance, as well as two *Peronia* slugs (Mollusca: Gastropoda: Onchidiidae) with a 5.4% pairwise distance. In the latter case, Chang et al. (2018) have shown, using an integrative taxonomic approach, that *Peronia* onchidiids in Singapore form a cryptic species complex. Cryptic diversity in the SIMP onchidiids were initially confirmed via BLAST matches in which IP0287 and IP0136 matched 99.8% and 99.9% to *Peronia* sp. 2 and *Peronia* “Singapore Clade”, respectively and corresponding morphological differences were found (Chang et al. 2018, Chan et al. 2019). More detailed morphological work is necessary to determine whether the *Ligia* species also belong to a species complex.

Indeed, DNA barcodes can help with species delimitation and cryptic species detection. DNA barcodes also allow for obtaining abundance and distribution information, but they tend to be of limited value for their original purpose, i.e. species identification, as only 36.2% of barcodes obtained here had species-level matches. A substantial number of our sequences that were matched to GenBank sequences at < 90% identity yielded only very tentative genus- or family-level identities. Even well-studied and common taxa such as molluscs, arthropods and fishes (e.g. *Hyselodoris*, *Dendrodoris*, *Ashtoret, Grapsus and Pomacentrus*) lacked barcodes in GenBank. Furthermore, in some taxa, the genus-level identities were of questionable accuracy. For example, amongst *Alpheus* shrimps, up to 20 specimens were recovered in the incorrect lineage with < 90% identity, with the closest match being a Caridea sp. at 82% to 88% sequence similarity (Suppl. material 3). The same situation was observed with synaptid sea cucumbers (Synaptidae) which lacked close matches to GenBank sequences. Some of these problems can be overcome by building a local database for Singapore that is supported by reliable morphological identifications.

The inadequacy of the barcode databases was particularly problematic for understudied groups such as annelids, platyhelminths, poriferans and zooplankton, such as chaetognaths. Amongst the 17 barcoded platyhelminth flatworm samples, for instance, all GenBank matches were < 88% in sequence identity and accurate only to the phylum level for 14 samples, while seven samples were assigned to the incorrect genus (see also Vanhove et al. 2013). Most platyhelminth flatworm studies use the 28S rDNA gene for phylogenetic analysis (Litvaitis and Rohde 1999, Litvaitis and Newman 2001, Bolaños et al. 2007, Bahia et al. 2017) and thus few COI sequences are represented in the global databases. Furthermore, the commonly used barcoding region *sensu* Folmer et al. (1994) does not overlap with the COI region that is informative for flatworms and thus lacks resolving power to delimit flatworm species (Vanhove et al. 2013) with the usual 3% barcoding threshold used across metazoans (Hebert et al. 2003). Amongst the zooplankton samples, we found up to 15 chaetognath MOTUs that could not be identified to species, either based on morphology or barcodes. Chaetognath species, such as *Krohnitta pacifica* and *Aidanosagitta crassa*, have been previously recorded in Singapore (Schmoker et al. 2014) but both species still lack COI barcodes in GenBank.

Overall, our study confirms that a substantial number of the sequences in the global databases are misidentified and that one should carefully distinguish between the use of the barcode sequences for, for example, obtaining distributional data and the use of barcode identification in the database. This is particularly important for understudied taxa (Zhang and Zhang 2014). For example, GenBank sequences of the copepod *Paracalanus aculeatus* had 100% matches to two different MOTUs which differed by an uncorrected p-distance of 9.3% (ZP011/ZP344 and ZP232 clusters). Conversely, a single cluster/sequence (e.g. ZP344) in our database matched at 100% to different *Paracalanus* species on GenBank, when compared to the BOLD database. Even well-studied taxa including fish, arthropods and molluscs, were not free from misidentification (e.g. Buhay 2009)—poor identity matches were obtained for our specimens of *Centrogenys vaigiensis*, *Ashtoret lunaris*, *Jorunna funebris*, for example, despite the presence of barcodes in GenBank that were filed under these names. We recommend that this gap be bridged by working with taxonomic experts in each pre-sorted group and, subsequently, supplementing local or global (i.e. GenBank, BOLD) databases with COI barcode sequences that are tagged with accurate species identities. This will facilitate future faunal identification studies.

For more than a decade, the COI locus has been popularised for barcoding a wide range of metazoan species (Hebert et al. 2003). Small intraspecific variation coupled with correspondingly large interspecific variation in the COI locus amongst most metazoan species sometimes yield a ‘barcoding gap’, which allows for accurate species identification using a generalised threshold of 3% between intra- and interspecific variabilities (Meyer and Paulay 2005, Meier et al. 2006). In reality, the distinctions between intra- and interspecific distances vary amongst taxa and the barcoding gap may not be present (Virgilio et al. 2010), especially in recently-diverged species (Meier et al. 2008, van Velzen et al. 2012). Such overlap can be caused by slow evolution of COI in some taxa (Hellberg 2006, Huang et al. 2008, Shearer et al. 2008), hybridisation events between sympatric species (Steinke et al. 2009, Ward et al. 2009) or by high sequence divergence, coupled with morphological stasis (Gómez et al. 2002). In particular, delimitation at the species and even genus level is difficult for many anthozoans, platyhelminth flatworms and some fish because of the lack of COI divergence (Steinke et al. 2009, Vanhove et al. 2013). For these groups, other markers need to be explored for DNA barcoding. To this end, combinations of different genes, including mitochondrial and nuclear loci, have been proposed and used for different groups of anthozoans (Huang et al. 2011) and the 28S rDNA for flatworms (Litvaitis and Rohde 1999, Litvaitis and Newman 2001). In anthozoans, species-level resolution is still limited due to frequent hybridisation (Quattrini et al. 2019), resulting in low interspecific divergence (Shearer and Coffroth 2008, Dohna and Kochzius 2015), though these markers are possibly useful at the genus level (Hsu et al. 2014). Other mitochondrial markers such as cytochrome *b* (Ward et al. 2005, Sevilla et al. 2007), 12S rRNA (Miya et al. 2015) and D-loop control region (Lee et al. 1995) have been suggested for use on barcoding fish species. Phylogenomic analyses in these groups are emerging (Egger et al. 2015, Lin et al. 2016, Marcionetti et al. 2018) and the data will help in the design of taxon-specific nuclear markers for future DNA barcoding work. With reducing costs of high-throughput DNA sequencing, multiple-gene DNA barcoding should become viable in the near future, which can help improve the accuracy of species identification.

The large number of species from many divergent lineages, examined here, would typically require a wide range of taxonomic expertise to sort the specimens into putative species, based on morphological data. This expertise was not readily available, so we use molecular tools for rapid and cost-effective species delimitation (Baloğlu et al. 2018, Wang et al. 2018) and occasionally for species identification if sequences can be matched to accurately-identified databases. Eventually, this barcoding exercise would follow the reverse workflow described in Wang et al. (2018), in which DNA barcodes act as precursory guides to direct the verification and evaluation by skilled taxonomists. While we have only managed to put names on 163 species, less than half of the > 365 species delimited here (Fig. 5), the data have already spawned collaborations with various specialists to focus on the more understudied fauna. In particular, taxonomic work elicited by our results is ongoing or recently accomplished for taxa such as corallimorpharians (Oh et al. 2019), corals (Poquita-Du et al. 2017), anemones (Yap et al. 2019) and onchidiid slugs (Chang et al. 2018, Chan et al. 2019), including potentially new species initially spotlighted by DNA barcodes generated here (Chang et al. 2018, Chan et al. 2019, Oh et al. 2019).

Our work here is only the beginning of further molecular ecological work in this biodiverse region. It follows recent, successful, large-scale biodiversity sampling exercises, such as the Moorea Biocode Project (Check 2006), which has helped pave the way for numerous other biodiversity studies and related applications, such as environmental DNA metabarcoding (Leray et al. 2011, Leray et al. 2013, Leray and Knowlton 2015), uncovering microbial diversity (McCliment et al. 2011), estimating biodiversity (Plaisance et al. 2009, Hubert et al. 2012), standardised reef biodiversity sampling (Leray and Knowlton 2015, Ransome et al. 2017), larval species identification (Hubert et al. 2010), designing metazoan-specific primers (Geller et al. 2013, Leray et al. 2013) and mapping entire island ecosystems (Achterberg et al. 2018) to designate genomic observatories (Field and Davies 2015). We can leverage on the DNA barcode database built for the SIMP to motivate the development of applications for better documenting species diversity in the region, thus strengthening the case for developing the marine park into a genomic observatory.

To understand why this is advantageous, we note that survey windows at the SIMP are limited in the intertidal areas by the tidal regime and in the subtidal by strong currents, so rapid and non-intrusive sampling methods such as environmental DNA (eDNA) would enable more regular surveys (Rees et al. 2014, Comtet et al. 2015). To this end, our DNA barcode database could serve as *the* reference library for matching and discovering species found in eDNA samples. Exploratory eDNA experiments based on 26 two-litre water samples collected from eight localities have led to the detection of > 500 metazoan MOTUs (Y.C.A. Ip, Y.C. Tay & J.J.M. Chang, unpublished data). Notably, 20 of these MOTUs were matched only to our local database and not the global databases GenBank and BOLD. This is remarkable, given the large repository of > 2.68 million COI sequences on GenBank. Indeed, the enhanced database resolution, resulting from thorough sampling at the SIMP, is crucial as eDNA has emerged as one of the main technology-driven tools for environmental monitoring and management.

## Conclusion

The collection and barcoding of marine animals at Sisters’ Islands and the surrounding islands began more than two years ago, at a time when these locations were designated Singapore’s first marine park. This is part of a larger initiative to make Singapore’s biodiversity identifiable, as well as to provide molecular identification tools for future work. Despite only a collection frequency of 34 times over a span of two years on foot and via SCUBA across a large 40-ha area and using only simple hand tools, nets and traps, our study managed to sample more than 365 species across a wide range of marine animals. A more systematic sampling approach, covering a larger area and using grabs, trawls, dredges and various nets will uncover greater diversity and more taxa, including infaunal and meiofaunal groups.

Being able to quantify and identify species diversity is important for many reasons, including the provision of a community baseline against which future surveys can be compared (Resh and Unzicker 1975). It is particularly critical for making better-informed decisions with respect to coastal reclamation and urban redevelopment (Chee et al. 2017), as well as monitoring the influx of introduced species. Records with geographic data are important for conservation when prioritising sites for protection (Anderson and Martínez-Meyer 2004) and for allowing biogeographic patterns at the regional and even global scales to be uncovered more precisely (Abell et al. 2008, Huang et al. 2018, Yip et al. 2019). Furthermore, our data comprise species records supported by linked photographic images and COI barcode sequences, potentially paving the way for more efficient biomonitoring applications such as eDNA testing. Fundamentally, these methods are revolutionising biodiversity studies, thus not only allowing scientists to discover species on Earth, but also allowing for more ready access to DNA-based identification via new small-sized sequencers whose use requires minimal amounts of laboratory equipment (Srivathsan et al. 2018).

## Supplementary Material

71066289-9045-5D41-81B5-2714CF1A33ED 10.3897/BDJ.7.e46833.suppl1 Supplementary material 1 Supplementary Review List S1a Data type: Compiled literature of SIMP biodiversity
Brief description: Existing published records documenting marine fauna at the Sisters' Islands Marine Park (SIMP), based on a keyword search of the literature. Literature keyword search was performed by entering keywords in the following order: “Sisters' Islands” or “Sisters' Islands Marine Park” or “Pulau Subar Laut” or “Pulau Subar Darat” or “Pulau Sakijang Bendera” or “Tanjong Hakim”.
File: oo_355445.pdf https://binary.pensoft.net/file/355445 Yin Cheong Aden Ip, Ywee Chieh Tay, Su Xuan Gan, Hui Ping Ang, Karenne Tun, Loke Ming Chou, Danwei Huang, Rudolf Meier

C200D20B-2DA4-5F64-802F-26FF72D3FEFE 10.3897/BDJ.7.e46833.suppl2 Supplementary material 2 Supplementary Review List S1b Data type: Compiled literature of SIMP COI barcodes
Brief description: Compiled literature of species records for which COI barcodes are available on GenBank. Species names from the compiled literature of SIMP biodiversity were searched on GenBank, returning 109 species with COI sequences that were sequenced elsewhere and these were compiled separately as a reference sequence database.
File: oo_341009.pdf https://binary.pensoft.net/file/341009 Yin Cheong Aden Ip, Ywee Chieh Tay, Su Xuan Gan, Hui Ping Ang, Karenne Tun, Loke Ming Chou, Danwei Huang, Rudolf Meier

E4FEDF17-288F-5DB2-A1CC-372292FB81EB 10.3897/BDJ.7.e46833.suppl3 Supplementary material 3 Supplementary Table S1a: SIMP macrofauna Data type: Metadata on macrofaunal specimens collected
Brief description: Information on macrofaunal specimen image availability, taxonomic information, genetic identity match to both GenBank and BOLD system databases, collection information, barcode availability, barcode length (long ≈ 658bp; short ≈ 313bp), LKCNHM Zoological Reference Collection (ZRC) catalogue numbers and GenBank numbers of all collected specimens.
File: oo_358747.xlsx https://binary.pensoft.net/file/358747 Yin Cheong Aden Ip, Ywee Chieh Tay, Su Xuan Gan, Hui Ping Ang, Karenne Tun, Loke Ming Chou, Danwei Huang, Rudolf Meier

515A5496-B14C-5251-BA26-56F9BB50FD39 10.3897/BDJ.7.e46833.suppl4 Supplementary material 4 Supplementary Table S1b: SIMP zooplankton Data type: Metadata on zooplankton specimens collected
Brief description: Information on zooplankton specimen image availability, taxonomic information, genetic identity match to both GenBank and BOLD system databases, collection information, barcode availability, barcode length (long ≈ 658bp; short ≈ 313bp), LKCNHM Zoological Reference Collection (ZRC) catalogue numbers and GenBank numbers of all collected specimens.
File: oo_358748.xlsx https://binary.pensoft.net/file/358748 Yin Cheong Aden Ip, Ywee Chieh Tay, Su Xuan Gan, Hui Ping Ang, Karenne Tun, Loke Ming Chou, Danwei Huang, Rudolf Meier

4349CC89-7475-5BCD-B2A5-7FFC4C6784AB 10.3897/BDJ.7.e46833.suppl5 Supplementary material 5 Supplementary Figure S1a Data type: Cluster dendrogram
Brief description: Cluster dendrogram based on percentage pairwise differences in COI for all 632 specimens with COI barcodes. Values at the nodes represent the percentage pairwise difference between two specimens. Taxon names on the branches represent taxonomic identities, based on morphological identification.
File: oo_341012.pdf https://binary.pensoft.net/file/341012 Yin Cheong Aden Ip, Ywee Chieh Tay, Su Xuan Gan, Hui Ping Ang, Karenne Tun, Loke Ming Chou, Danwei Huang, Rudolf Meier

21F7C624-280B-50CD-ACF5-B9475CC42219 10.3897/BDJ.7.e46833.suppl6 Supplementary material 6 Supplementary Figure S1b Data type: Cluster dendrogram
Brief description: Cluster dendrogram based on percentage pairwise differences in COI for 304 specimens (excluding IP0303) with species-level identification. Values at the nodes represent the percentage pairwise difference between two specimens. Taxon names on the branches represent taxonomic identities, based on morphological identification.
File: oo_341013.pdf https://binary.pensoft.net/file/341013 Yin Cheong Aden Ip, Ywee Chieh Tay, Su Xuan Gan, Hui Ping Ang, Karenne Tun, Loke Ming Chou, Danwei Huang, Rudolf Meier

## Figures and Tables

**Figure 1a.**
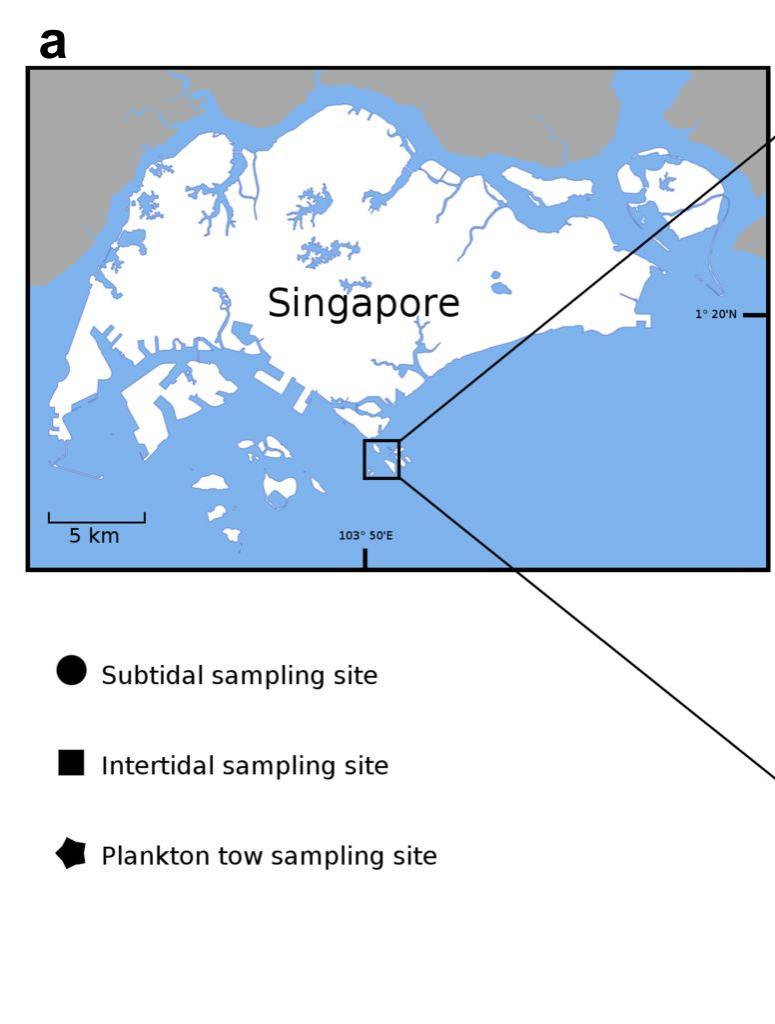
Singapore

**Figure 1b.**
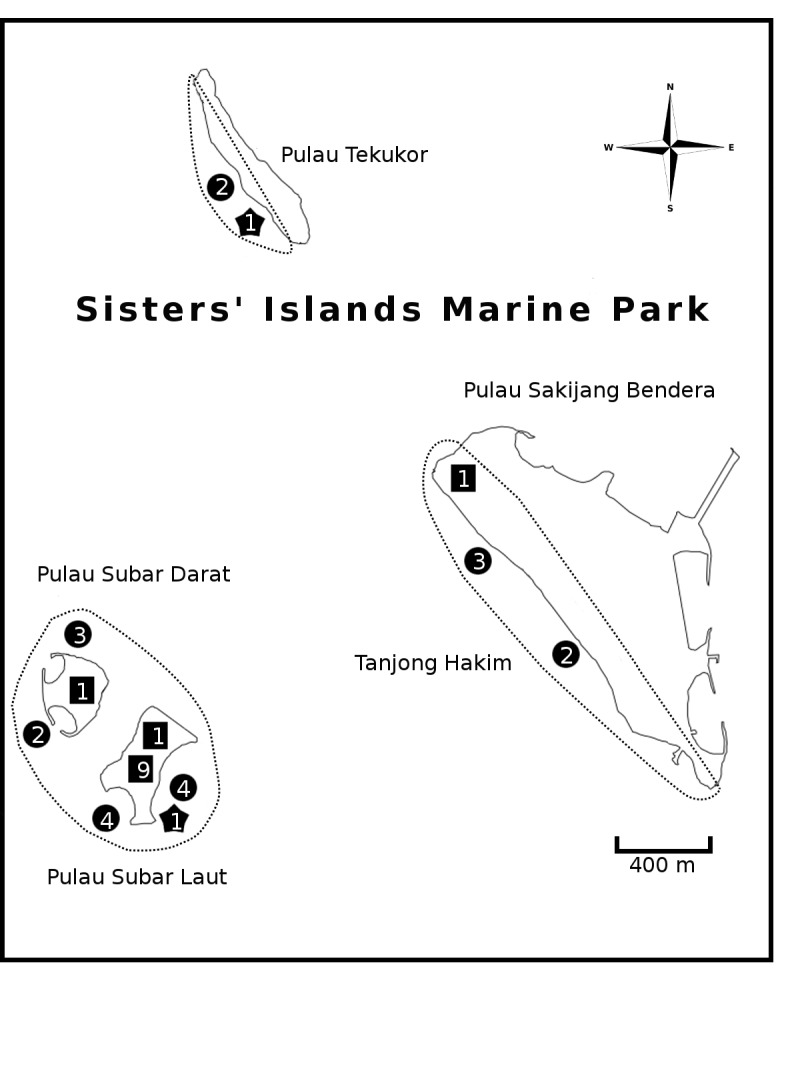
Sisters’ Islands Marine Park

**Figure 2a.**
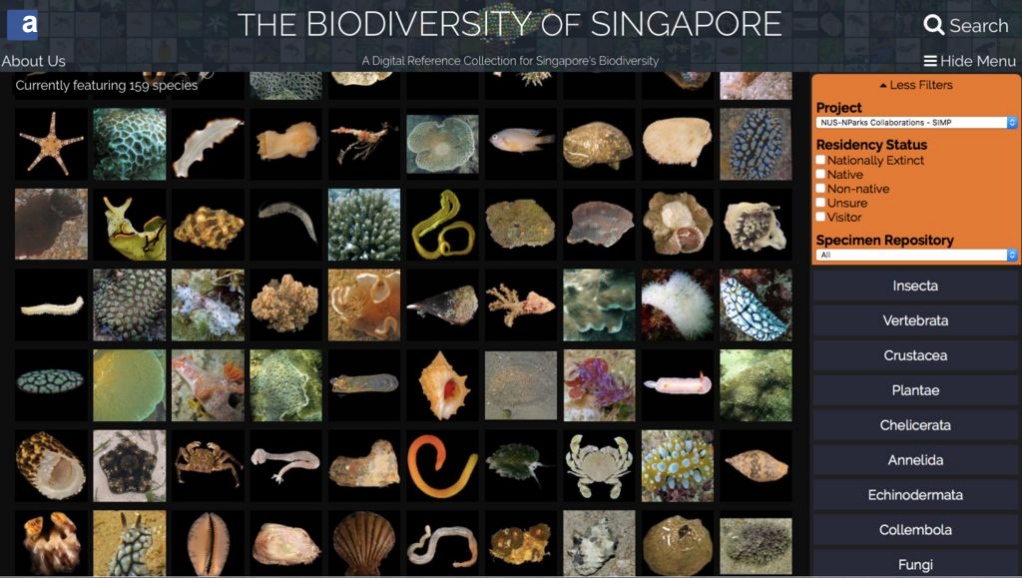
Animal taxa are organised by taxonomic identity

**Figure 2b.**
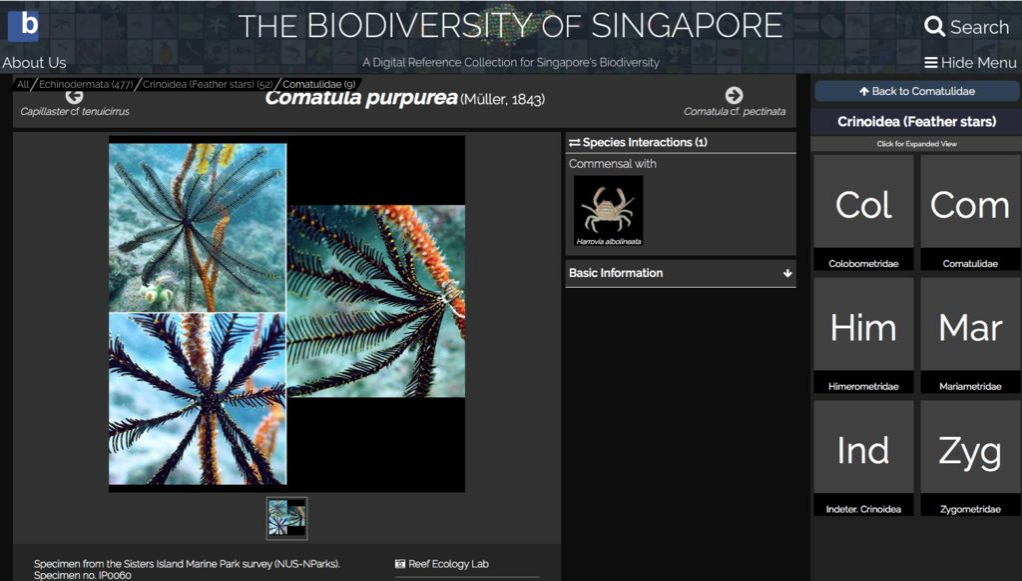
A thumbnail is available for each taxon that links to an individual web page with more detailed information and/or photos

**Figure 3a.**
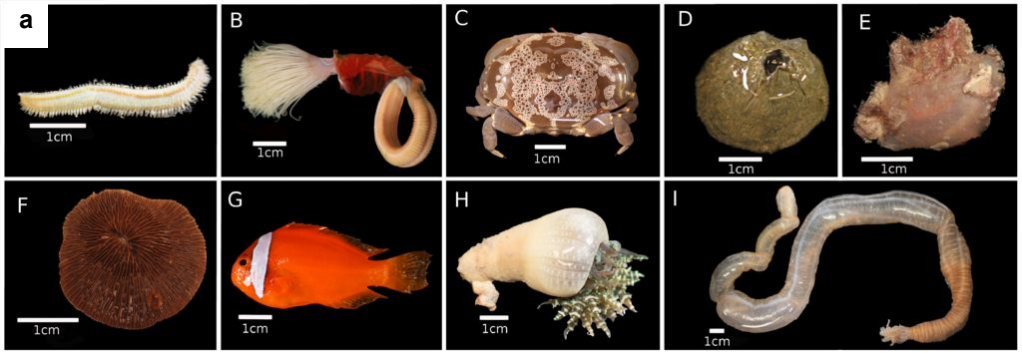
(A, B) Phylum Annelida. (A) *Eurythoe* sp., IP0133; (B) *Protula* sp., IP0315. (C, D) Phylum Arthropoda. (C) *Atergatis floridus*, IP0450; (D) *Tetraclita squamosa*, IP0106 [ZRC 2017.1114]. (E, G) Phylum Chordata. (E) F. Pyuridae, IP0070; (G) *Amphiprion frenatus*, IP0479. (F, H) Phylum Cnidaria. (F) *Lithophyllon scabra*, IP0336; (H) *Phymanthus* sp., IP0122 [ZRC.CNI.1249].

**Figure 3b.**
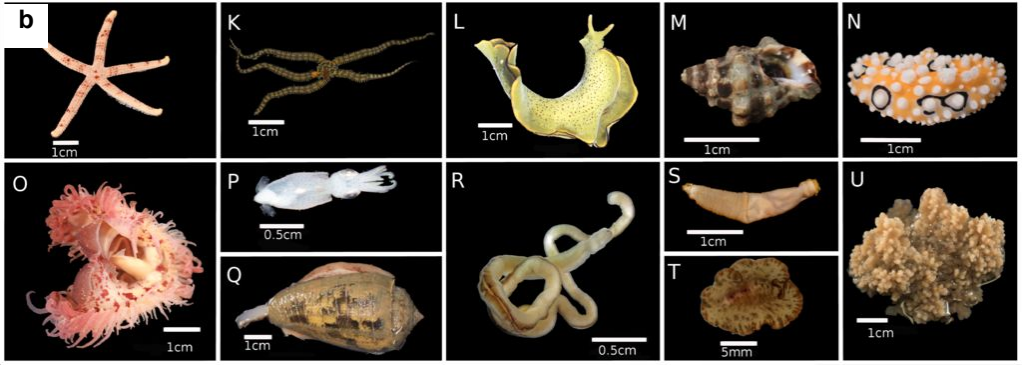
(I, J, K) Phylum Echinodermata. (I) F. Synaptidae, IP0142; (J) *Nepanthia* sp., IP0321 [ZRC.ECH.1253]; (K) *Ophiactis* sp., IP0207 [ZRC.ECH.1243]. (L, M, N, O, P, Q) Phylum Mollusca. (L) *Elysia ornata*, IP0269 [ZRC.MOL.010720]; (M) *Tenguella* sp., IP0096 [ZRC.MOL.010698]; (N) *Phyllidia ocellata*, IP0192; (O) *Limaria* sp., IP0263 [ZRC.MOL.010718]; (P) *Idiosepius pygmaeus*, IP0115 [ZRC.MOL.010704]; (Q) F. Conidae, IP0144. (R) Phylum Nemertea, IP0108 [ZRC.MIS.0006]. (S) Phylum Sipuncula, IP0085 [ZRC.SIP.0030]. (T) Phylum Platyhelminthes. *Pseudobiceros damawan*, IP0447. (U) Phylum Porifera, IP0396.

**Figure 4a.**
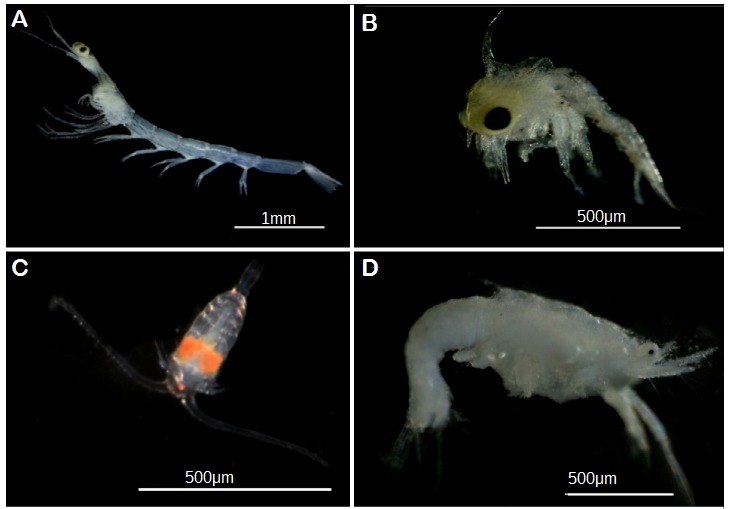
(A, B, C, D, G) Phylum Arthropoda. (A) F. Luciferidae, ZP024 (Naomi et al. 2006); (B) Order Decapoda, ZP328; (C) Order Calanoida, unsequenced live copepod; (D) *Acetes indicus*, ZP332; (G) *Tetraclita singaporensis*, ZP277;

**Figure 4b.**
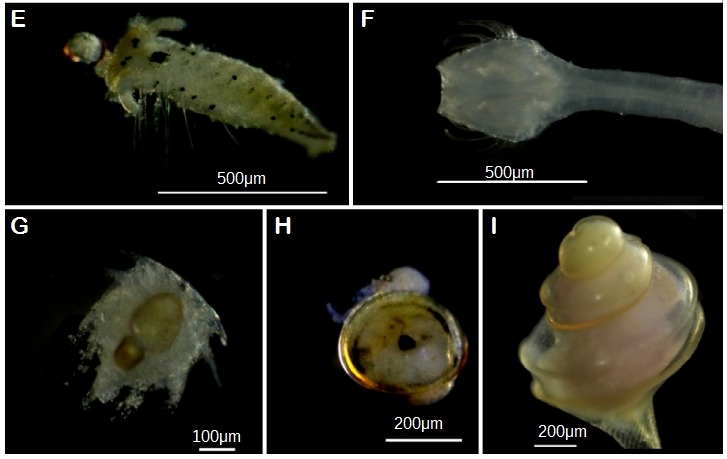
(E) Phylum Annelida. *Polydora aura*, ZP288; (F) Phylum Chaetognatha. F. Sagittidae, ZP278; (H, I) Phylum Mollusca. (H) *Dendostrea frons*, ZP016; (I) F. Turridae, ZP312.

**Figure 5a.**
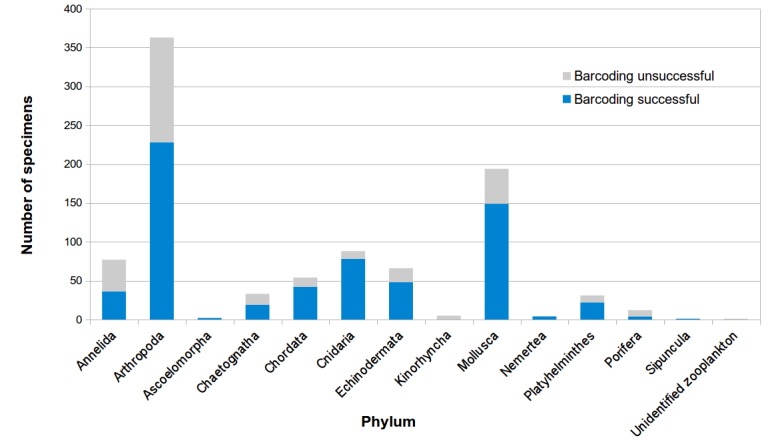
Barcoding success across specimens collected. Numbers of specimens for which COI barcoding was successful and not successful, are indicated by blue and grey shades, respectively.

**Figure 5b.**
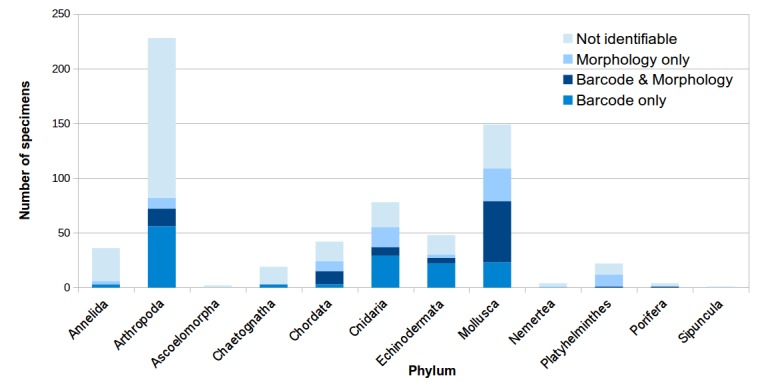
Identification success to species level for specimens, for which barcodes were successfully obtained. Numbers of specimens identified based only on morphology, only on barcode matches or via both methods which were congruent, are represented by different shades of blue.

## Hosting institution

National University of Singapore
