# Supplementary material for: From marine park to future genomic observatory? Enhancing marine biodiversity assessments using a biocode approach
Source: Biodivers Data J. 2019 Dec 10;7:e46833. doi: 10.3897/BDJ.7.e46833 (PMC6917626; doi:10.3897/BDJ.7.e46833)
Supplement: Supplementary material 1 — Supplementary Review List S1a [file bdj-07-e46833-s001.pdf]

Review list S1a. Compiled literature of SIMP biodiversity

Literature keyword search was done by entering keywords in the following order: “Sisters' Islands” or “Sisters' Islands Marine Park” or “Pulau Subar Laut” or “Pulau Subar Darat” or “Pulau Sakijang Bendera” or “Tanjong Hakim”.

Existing published records documenting marine fauna at the SIMP, based on a keyword search in the literature. This was performed by entering keywords in the following order: “Sisters' Islands” or “Sisters' Islands Marine Park” or “Pulau Subar Laut” or “Pulau Subar Darat” or “Pulau Tekukor” or “Pulau Sakijang Bendera”.

1. Bioblitz coral survey data, National Parks Board
2. Census of Marine Life, Jesse H. Ausubel
3. Bolaños, D. M., Gan, B. Q., & Ong, R. S. (2016). First records of pseudocerotid flatworms (Platyhelminthes: Polycladida: Cotylea) from Singapore: A taxonomic report with remarks on colour variation. Raffles Bulletin of Zoology.
4. Bruce, N. L., & Wong, H. P. S. (2015). An overview of the marine Isopoda (Crustacea) of Singapore. Raffles Bulletin of Zoology.
5. Goh, B. P., & Lim, D. Y. (2015). Distribution and abundance of sea urchins in Singapore reefs and their potential ecological impacts on macroalgae and coral communities. Ocean Science Journal, 50(2), 211-219.
6. Guest, J. R., Todd, P. A., Goh, E., Sivalonganathan, B. S., & Reddy, K. P. (2008). Can giant clam (*Tridacna squamosa*) populations be restored on Singapore's heavily impacted coral reefs. Aquatic Conservation: Marine and Freshwater Ecosystems, 18(5), 570-579.
7. Hoeksema, B. W., & Koh, E. G. (2009). Depauperation of the mushroom coral fauna (Fungiidae) of Singapore (1860s–2006) in changing reef conditions. Raffles Bull Zool Suppl, 22, 91-101.
8. Huang, D., Meier, R., Todd, P. A., & Chou, L. M. (2008). Slow mitochondrial COI sequence evolution at the base of the metazoan tree and its implications for DNA barcoding. Journal of Molecular Evolution, 66(2), 167-174.
9. Huang, D., Tun, K. P., Chou, L. M., & Todd, P. A. (2009). An inventory of zooxanthellate scleractinian corals in Singapore, including 33 new records. Raffles Bulletin of Zoology, 22, 69-80.
10. Hung, S. M., Chong, K. Y., Yee, A. T., Lim, R. C., Loh, J. W., Neo, L., ... & Tong, C. H. (2017). The vascular plant flora and vegetation of the islands associated with Singapore’s first Marine Park (I): The Sisters’ Islands.
11. Lee, A. C., Tan, K. S., & Sin, T. M. (2009). Intertidal assemblages on coastal defence structures in Singapore I: a faunal study. Raffles Bulletin of Zoology, 22, 237-254.
12. Low, J. K., Tanzil, J. I. T., & Jaafar, Z. (2013). More noteworthy fishes observed in the Singapore Straits. Nature in Singapore, 6, 31-37.
13. Low, J., & Chou, L. M. (2013). Sargassum in Singapore: What, Where and When. Taxonomy of Southeast Asian Seaweeds II. Institute of Ocean and Earth Sciences, University of Malaya Monograph Series, 15, 219-235.
14. McKenzie, L. J., Yaakub, S. M., Tan, R., Seymour, J., & Yoshida, R. L. (2016). Seagrass habitats of Singapore: Environmental drivers and key processes. Raffles

Bulletin of Zoology.
15. Neo, M. L., & Todd, P. A. (2012). Population density and genetic structure of the giant clams *Tridacna crocea* and *T. squamosa* on Singapore’s reefs. Aquatic Biology, 14(3), 265-275.
16. Ng, H. H., & Lim, K. K. P. (2014). A preliminary checklist of the cardinalfishes (Actinopterygii: Gobiiformes: Apogonidae) of Singapore. Check List, 10(5), 1061-1070.17. Ng, C. S. L., Toh, T. C., & Chou, L. M. (2017). Artificial reefs as a reef restoration strategy in sediment-affected environments: Insights from long-term monitoring. Aquatic Conservation: Marine and Freshwater Ecosystems.
18. Ong, C. W., Reimer, J. D., & Todd, P. A. (2013). Morphologically plastic responses to shading in the zoanthids *Zoanthus sansibaricus* and *Palythoa* tuberculosa. Marine Biology, 160(5), 1053-1064.
19. Siang, T. K. (2012). Observations on the subtidal fouling community on jetty pilings in the Southern Islands of Singapore.
20. Tan, S. K., & Clements, R. (2008). Taxonomy and distribution of the Neritidae (Mollusca: Gastropoda) in Singapore. Zoological studies, 47(4), 481-494.
21. Tan, K. S., Koh, K. S., Ng, J. Y., Goh, L. (2016). The Comprehensive Marine Biodiversity Survey Singapore Strait International Workshop 2013. Raffles Bulletin of Zoology Supplement 34:1-7.
22. Teo, S. Y., Ng, C. S. L., Suen, S. M., Lok, A. F. S. L., & Ng, P. X. (2010). Notes on the sea cucumber, *Afrocucumis africana* (Semper, 1868)(Holothuroidea: Dendrochirotida: Sclerodactylidae) in Singapore. Nature in Singapore, 3, 65-68.
23. Tilbrook, K. J., & Gordon, D. P. (2016). Checklist of Singaporean Bryozoa and Entoprocta. Raffles Bulletin of Zoology.
