# Supplementary material for: From marine park to future genomic observatory? Enhancing marine biodiversity assessments using a biocode approach
Source: Biodivers Data J. 2019 Dec 10;7:e46833. doi: 10.3897/BDJ.7.e46833 (PMC6917626; doi:10.3897/BDJ.7.e46833)
Supplement: Supplementary material 2 — Supplementary Review List S1b [file bdj-07-e46833-s002.pdf]

S/N	Taxa	Literature	GenBank COI availability	GenBank accession no.	Genus	Species
1	Scleractinia	BioBlitz	yes	EU371658	*Acanthastrea*	*echinata*
2	Scleractinia	BioBlitz	yes	KX664149	*Acropora*	*secale*
3	Scleractinia	BioBlitz	yes	KX664157	*Acropora*	*valida*
4	Scleractinia	BioBlitz	no	-	*Astreopora*	*expansa*
5	Scleractinia	BioBlitz	yes	AB441253	*Astreopora*	*myriophthalma*
6	Scleractinia	BioBlitz	no	-	*Bernardpora*	*stutchburyi*
7	Scleractinia	BioBlitz	yes	KJ666421	*Coelastrea*	*aspera*
8	Scleractinia	BioBlitz	yes	KJ666425	*Coelastrea*	*palauensis*
9	Scleractinia	BioBlitz	yes	AM494859	*Coscinaraea*	*columna*
10	Scleractinia	BioBlitz	yes	EU149879	*Ctenactis*	*echinata*
11	Scleractinia	BioBlitz	yes	FJ345415	*Cyphastrea*	*chalcidicum*
12	Scleractinia	BioBlitz	yes	FJ345416	*Cyphastrea*	*microphthalma*
13	Scleractinia	BioBlitz	yes	FJ345417	*Cyphastrea*	*serailia*
14	Scleractinia	BioBlitz	yes	EU371660	*Diploastrea*	*heliopora*
15	Scleractinia	BioBlitz	no	-	*Dipsastraea*	*favus*
16	Scleractinia	BioBlitz	no	-	*Dipsastraea*	*lizardensis*
17	Scleractinia	BioBlitz	no	-	*Dipsastraea*	*matthaii*
18	Scleractinia	BioBlitz	no	-	*Dipsastraea*	*maxima*
19	Scleractinia	BioBlitz	no	-	*Dipsastraea*	*mirabilis*
20	Scleractinia	BioBlitz	no	-	*Dipsastraea*	*pallida*
21	Scleractinia	BioBlitz	no	-	*Dipsastraea*	*speciosa*
22	Scleractinia	BioBlitz	yes	LT605252	*Echinophyllia*	*aspera*
23	Scleractinia	BioBlitz	yes	FJ345418	*Echinopora*	*gemmacea*
24	Scleractinia	BioBlitz	yes	HQ203253	*Echinopora*	*horrida*
25	Scleractinia	BioBlitz	yes	FJ345419	*Echinopora*	*lamellosa*
26	Scleractinia	BioBlitz	yes	AB441204	*Euphyllia*	*ancora*
27	Scleractinia	BioBlitz	yes	AB441203	*Euphyllia*	*divisa*
28	Scleractinia	BioBlitz	yes	EU371687	*Favites*	*abdita*
29	Scleractinia	BioBlitz	yes	EU371688	*Favites*	*chinensis*
30	Scleractinia	BioBlitz	yes	EU371692	*Favites*	*complanata*
31	Scleractinia	BioBlitz	yes	EU371693	*Favites*	*flexuosa*
32	Scleractinia	BioBlitz	yes	HE654592	*Favites*	*halicora*
33	Scleractinia	BioBlitz	yes	FJ345429	*Favites*	*pentagona*
34	Scleractinia	BioBlitz	yes	EU371719	*Favites*	*valenciennesi*
35	Scleractinia	BioBlitz	no	-	*Favites*	*vasta*
36	Scleractinia	BioBlitz	yes	JQ920457	*Galaxea*	*astreata*
37	Scleractinia	BioBlitz	yes	HQ420829	*Galaxea*	*fascicularis*
38	Scleractinia	BioBlitz	yes	EU371697	*Goniastrea*	*edwardsi*
39	Scleractinia	BioBlitz	yes	FJ345434	*Goniastrea*	*pectinata*
40	Scleractinia	BioBlitz	yes	AB748769	*Goniopora*	*cf.lobata*
41	Scleractinia	BioBlitz	yes	JQ920447	*Goniopora*	*columna*
42	Scleractinia	BioBlitz	yes	LT547037	*Goniopora*	*djiboutiensis*
43	Scleractinia	BioBlitz	yes	LT547086	*Goniopora*	*minor*
44	Scleractinia	BioBlitz	yes	LT547161	*Goniopora*	*stokesi*
45	Scleractinia	BioBlitz	yes	LC191478	*Herpolitha*	*limax*
46	Scleractinia	BioBlitz	no	-	*Hydnophora*	*cf.microconos*
47	Scleractinia	BioBlitz	yes	HE654621	*Hydnophora*	*exesa*
48	Scleractinia	BioBlitz	yes	HE654609	*Leptastrea*	*pruinosa*
49	Scleractinia	BioBlitz	yes	EU371705	*Leptoria*	*phrygia*
50	Scleractinia	BioBlitz	yes	LC191480	*Lithophyllon*	*concinna*
51	Scleractinia	BioBlitz	no	-	*Lithophyllon*	*repanda*
52	Scleractinia	BioBlitz	yes	LC191482	*Lithophyllon*	*scabra*
53	Scleractinia	BioBlitz	yes	EU149887	*Lithophyllon*	*undulatum*
54	Scleractinia	BioBlitz	no	-	*Lobophyllia*	*agaricia*
55	Scleractinia	BioBlitz	yes	AB117241	*Lobophyllia*	*corymbosa*
56	Scleractinia	BioBlitz	yes	AB117240	*Lobophyllia*	*hemprichii*
57	Scleractinia	BioBlitz	no	-	*Lobophyllia*	*radians*
58	Scleractinia	BioBlitz	no	-	*Lobophyllia*	*recta*
59	Scleractinia	BioBlitz	no	-	*Lobophyllia*	*valenciennesi*

S/N	Taxa	Literature	GenBank COI availability	GenBank accession no.	Genus	Species
60	Scleractinia	BioBlitz	yes	KJ666509	*Merulina*	*ampliata*
61	Scleractinia	BioBlitz	yes	KJ666519	*Merulina*	*scabricula*
62	Scleractinia	BioBlitz	no	-	*Montipora*	*mollis*
63	Scleractinia	BioBlitz	no	-	*Montipora*	*monasteriata*
64	Scleractinia	BioBlitz	no	-	*Montipora*	*verrucosa*
65	Scleractinia	BioBlitz	yes	HQ203294	*Mycedium*	*elephantotus*
66	Scleractinia	BioBlitz	yes	FJ345438	*Oulophyllia*	*bennettae*
67	Scleractinia	BioBlitz	yes	EU371721	*Oulophyllia*	*crispa*
68	Scleractinia	BioBlitz	yes	AB117256	*Oxypora*	*lacera*
69	Scleractinia	BioBlitz	yes	LK934498	*Pachyseris*	*speciosa*
70	Scleractinia	BioBlitz	yes	KJ666443	*Paragoniastrea*	*australensis*
71	Scleractinia	BioBlitz	yes	LT630832	*Pavona*	*cactus*
72	Scleractinia	BioBlitz	yes	LT630840	*Pavona*	*decussata*
73	Scleractinia	BioBlitz	yes	LT630848	*Pavona*	*explanulata*
74	Scleractinia	BioBlitz	yes	JQ920448	*Pavona*	*frondifera*
75	Scleractinia	BioBlitz	yes	HQ203298	*Pectinia*	*alcicornis*
76	Scleractinia	BioBlitz	no	-	*Pectinia*	*crassa*
77	Scleractinia	BioBlitz	yes	HQ203300	*Pectinia*	*lactuca*
78	Scleractinia	BioBlitz	yes	HQ203301	*Pectinia*	*paeonia*
79	Scleractinia	BioBlitz	yes	FJ345440	*Platygyra*	*daedalea*
80	Scleractinia	BioBlitz	yes	EU371723	*Platygyra*	*pini*
81	Scleractinia	BioBlitz	yes	FJ345442	*Platygyra*	*sinensis*
82	Scleractinia	BioBlitz	yes	EU371722	*Platygyra*	*verweyi*
83	Scleractinia	BioBlitz	yes	KU762009	*Plerogyra*	*sinuosa*
84	Scleractinia	BioBlitz	yes	HQ203247	*Plesiastrea*	*versipora*
85	Scleractinia	BioBlitz	yes	LC191471	*Pleuractis*	*moluccensis*
86	Scleractinia	BioBlitz	yes	EU149912	*Pleuractis*	*paumotensis*
87	Scleractinia	BioBlitz	no	-	*Pocillopora*	*acuta*
88	Scleractinia	BioBlitz	yes	AM494871	*Podabacia*	*crustacea*
89	Scleractinia	BioBlitz	yes	EU149915	*Polyphyllia*	*talpina*
90	Scleractinia	BioBlitz	no	-	*Porites*	*australiensis*
91	Scleractinia	BioBlitz	yes	LT558154	*Porites*	*lobata*
92	Scleractinia	BioBlitz	yes	LT558159	*Porites*	*lutea*
93	Scleractinia	BioBlitz	yes	LT558165	*Porites*	*rus*
94	Scleractinia	BioBlitz	yes	AM494850	*Psammocora*	*contigua*
95	Scleractinia	BioBlitz	yes	FM865874	*Psammocora*	*haimiana*
96	Scleractinia	BioBlitz	yes	FM865878	*Psammocora*	*nierstraszi*
97	Scleractinia	BioBlitz	yes	AM494867	*Pseudosiderastrea*	*tayamai*
98	Scleractinia	BioBlitz	no	-	*Stylocoeniella*	*armata*
99	Scleractinia	BioBlitz	yes	HQ203306	*Trachyphyllia*	*geoffroyi*
100	Scleractinia	BioBlitz	yes	HG965346	*Tubastraea*	*coccinea*
101	Scleractinia	BioBlitz	yes	HG965281	*Tubastraea*	*diaphana*
102	Scleractinia	BioBlitz	yes	HG965350	*Tubastraea*	*micranthus*
103	Scleractinia	BioBlitz	yes	HG965360	*Turbinaria*	*mesenterina*
104	Scleractinia	BioBlitz	yes	HG965364	*Turbinaria*	*peltata*
105	Scleractinia	BioBlitz	yes	HG965366	*Turbinaria*	*reniformis*
106	Scleractinia	Huang et al (2008)	yes	EU371696	*Goniastrea*	*australensis*
107	Scleractinia	Huang et al (2008)	yes	EU371694	*Favites*	*paraflexuosa*
108	Bivalvia	Neo & Todd (2012)	yes	JN392066	*Tridacna*	*crocea*
109	Bivalvia	Neo & Todd (2012)	yes	JN392020	*Tridacna*	*squamosa*
