# Supplementary material for: From marine park to future genomic observatory? Enhancing marine biodiversity assessments using a biocode approach
Source: Biodivers Data J. 2019 Dec 10;7:e46833. doi: 10.3897/BDJ.7.e46833 (PMC6917626; doi:10.3897/BDJ.7.e46833)
Supplement: Supplementary material 5 — Supplementary Figure S1a [file bdj-07-e46833-s005.pdf]

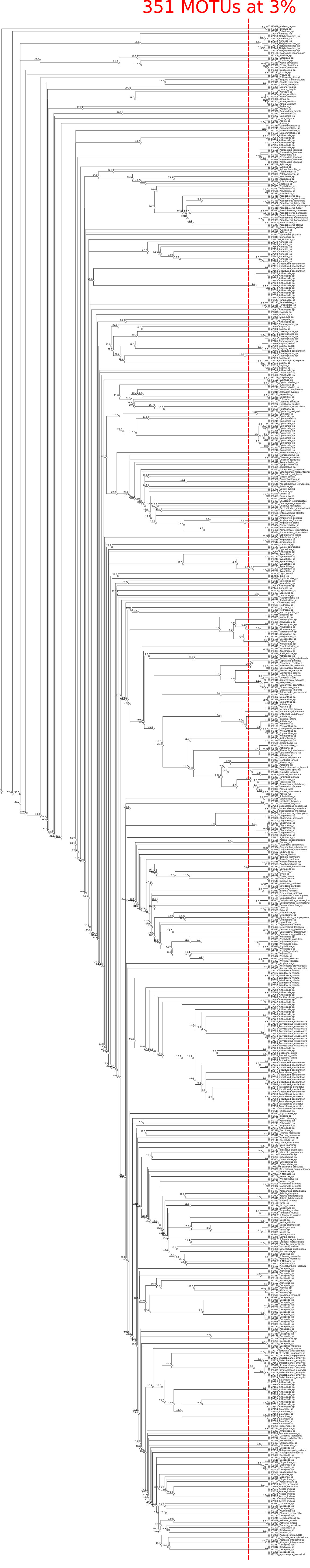

Figure S1. Cluster dendrogram based on percentage pairwise differences in COI.
a. Numerical values at the nodes represent the percentage pairwise difference between two specimens. Taxa names on the branches represent taxonomical identities based on morphological identifications. This includes all 632 specimens with COI barcodes. Taxonomical identities are indicated based on both morphological identifications where available.
