# Supplementary material for: From marine park to future genomic observatory? Enhancing marine biodiversity assessments using a biocode approach
Source: Biodivers Data J. 2019 Dec 10;7:e46833. doi: 10.3897/BDJ.7.e46833 (PMC6917626; doi:10.3897/BDJ.7.e46833)
Supplement: Supplementary material 6 — Supplementary Figure S1b [file bdj-07-e46833-s006.pdf]

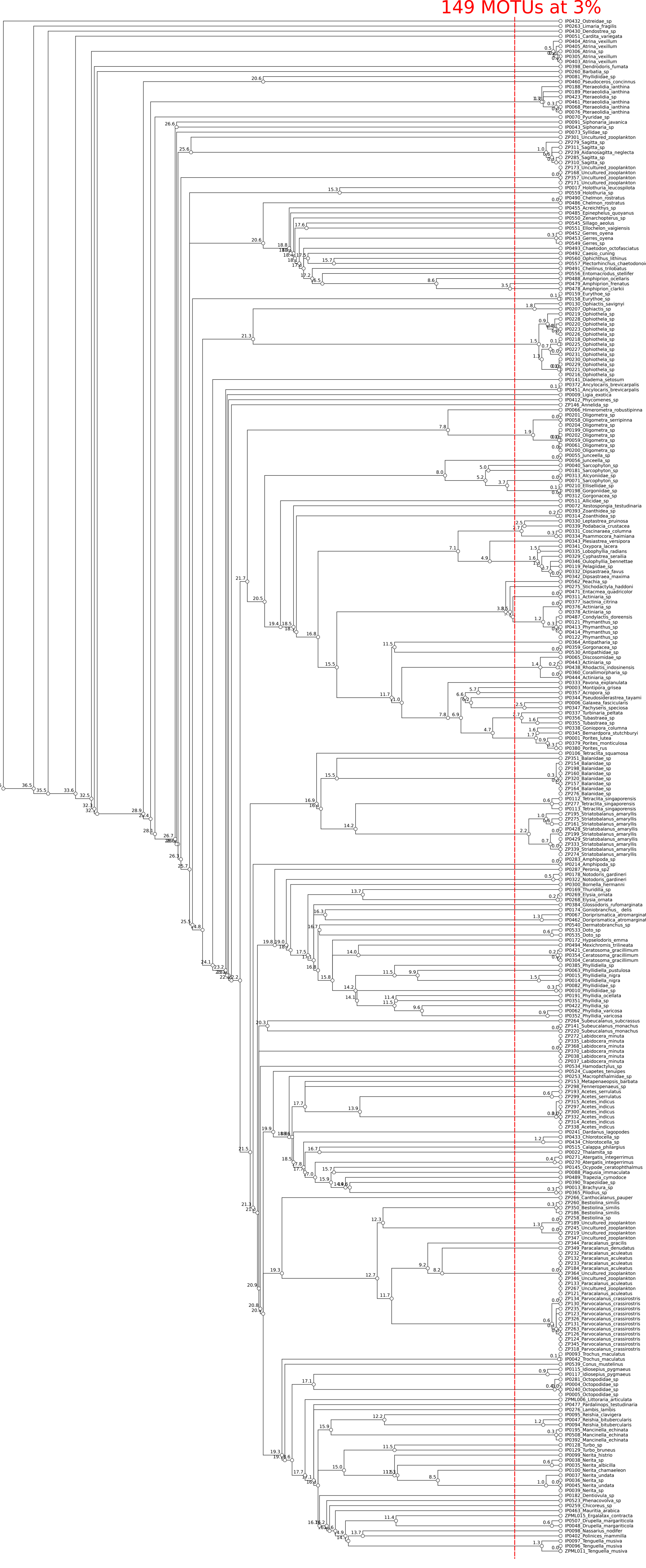

Figure S1. Cluster dendrogram based on percentage pairwise differences in COI.
b. Numerical values at the nodes represent the percentage pairwise difference between two specimens. Taxa names on the branches represent taxonomical identities based on morphological identifications. Taxonomical identities are indicated based on both morphological identifications where available. This only includes the 304 (excluding IP0303) specimens with species-level identifications.
